# PEO based polymer-ceramic hybrid solid electrolytes: a review

**DOI:** 10.1186/s40580-020-00252-5

**Published:** 2021-01-10

**Authors:** Jingnan Feng, Li Wang, Yijun Chen, Peiyu Wang, Hanrui Zhang, Xiangming He

**Affiliations:** 1 Department of Applied Physics and Applied Mathematics, Columbia University, New York, NY 10027 USA; 2 Institute of Nuclear and New Energy Technology, Tsinghua University, Beijing, 100084 China

**Keywords:** Solid electrolyte, Polymer electrolyte, Polyethylene oxide, Ceramic, Nanoparticle

## Abstract

Compared with traditional lead-acid batteries, nickel–cadmium batteries and nickel-hydrogen batteries, lithium-ion batteries (LIBs) are much more environmentally friendly and much higher energy density. Besides, LIBs own the characteristics of no memory effect, high charging and discharging rate, long cycle life and high energy conversion rate. Therefore, LIBs have been widely considered as the most promising power source for mobile devices. Commonly used LIBs contain carbonate based liquid electrolytes. Such electrolytes own high ionic conductivity and excellent wetting ability. However, the use of highly flammable and volatile organic solvents in them may lead to problems like leakage, thermo runaway and parasitic interface reactions, which limit their application. Solid polymer electrolytes (SPEs) can solve these problems, while they also bring new challenges such as poor interfacial contact with electrodes and low ionic conductivity at room temperature. Many approaches have been tried to solve these problems. This article is divided into three parts to introduce polyethylene oxide (PEO) based polymer-ceramic hybrid solid electrolyte, which is one of the most efficient way to improve the performance of SPEs. The first part focuses on polymer-lithium salt (LiX) matrices, including their ionic conduction mechanism and impact factors for their ionic conductivity. In the second part, the influence of both active and passive ceramic fillers on SPEs are reviewed. In the third part, composite SPEs’ preparation methods, including solvent casting and thermocompression, are introduced and compared. Finally, we propose five key points on how to make composite SPEs with high ionic conductivity for reference.

## Introduction

Lithium ion batteries (LIBs) are widely used in phones, computers and other mobile devices owing to their high specific energy, good capability, good cycle performance and environmentally friendly property [1–3]. Although traditional carbonate based liquid electrolytes have high ionic conductivity under normal temperature, the organic solvent contained has the potential danger of leakage and combustion, which may cause severe safety issues. Solid-state electrolytes, both inorganic solid electrolytes and solid polymer electrolytes, can overcome these shortages [4]. The research on solid-state ionic conductors can trace back to 1834. However, the real threshold of studies on solid-state electrolytes is generally believed to be 1960s when Takahashi et al. [5] found the silver ionic conductivity of Ag_3_SI (about 10^−2^ S/cm at ambient temperature). These ceramic materials own excellent ionic conductivity, while they have fatal defects that their rigid and brittle bodies will lead to bad contact with electrodes and bring great difficulties to processing. Therefore, the focus of research gradually shifted from inorganic materials to organic materials which own good flexibility and processability. In 1973, Fenton et al. [6] reported the transport of ions in polyethylene oxide (PEO)-alkali metal salts complexation, which started a new chapter of researches on solid polymer electrolytes (SPEs). After that, Armand et al. [7] reported that the ionic conductivity of such complexations could reach 10^−5^ S/cm in temperature range between 40 and 60 °C, indicating that SPEs might be used under room temperature. Since then, the research on SPEs has become the most popular part in related fields.

Compared with traditional liquid electrolytes, SPEs can not only alleviate the danger of flammability and possible side reactions with electrodes, but also retain the excellent adhesion and film-forming properties of polymers. Moreover, the solid-state electrolyte membranes are supposed to suppress the growing of lithium dendrite, which can further ensure the safety of the batteries during the charging and discharging process. These characteristics determine that SPEs have a promising future. However, SPEs have not yet reached the practical accessibility due to their huge interfacial resistance and low bulk conductivity (10^−7^ S/cm) at ambient temperature [8, 9]. To solve these problems, polymer/liquid hybrid [10], polymer/polymer hybrid and polymer/ceramic hybrid were developed [11]. The polymer/liquid hybrid is to form a gel-type polymer electrolyte by adding a small amount of liquid plasticizer, such as ethylene carbonate (EC), propylene carbonate (PC) and dimethyl carbonate (DMC) into the electrolyte. In such hybrid, the liquid phase plays an important role in wetting, reducing the interfacial resistance and directly increasing the ionic conductivity. Nevertheless, such improvement is at the expense of all-solid-state properties of SPEs, which may reduce the mechanical strength of the electrolytes and cause similar problems to liquid electrolytes. The polymer/polymer hybrid is formed by combining different kinds of polymers so that the hybrid can obtain the advantages of each one. As an example, the ionic conductivity of polystyrene (PS)-PEO-PS [12, 13] hybrid system can reach 2.3 × 10^−4^ S/cm at 60 °C, which is better than the performance of single PEO-based SPEs. But the ionic conductivity is still not high enough for daily use under normal temperature. The polymer/ceramic hybrid is to add inorganic fillers into the electrolytes. These fillers can be divided to two categories due to their own nature. The first are passive fillers for there are no Li ions in themselves and thus they do not directly participate into the transport process of Li^+^. They can improve the ionic conductivity of SPEs mainly due to the complicated structure of polymer-passive filler interfaces, which results in the suppression of recrystallization of PEO, more free-Li^+^ and fast ion transport channels. The fillers in second kind are active, which contain Li ions in their bodies. As a result, Li^+^ can transport through PEO body, PEO-active filler interfaces and active fillers’ bodies. The ionic conductivity of the whole SPEs get enhanced in this way. Besides, the mechanical strength can get better by doping with hard ceramic particles [14] and the interfacial stability between electrodes and electrolytes are supposed to be improved mainly due to the water-scavenging effect of these particles [15–18]. Consequently, polymer-ceramics hybrid become one of the most effective way to improve the performance of SPEs.

## Polymer-LiX matrices

**Fig. 1:**
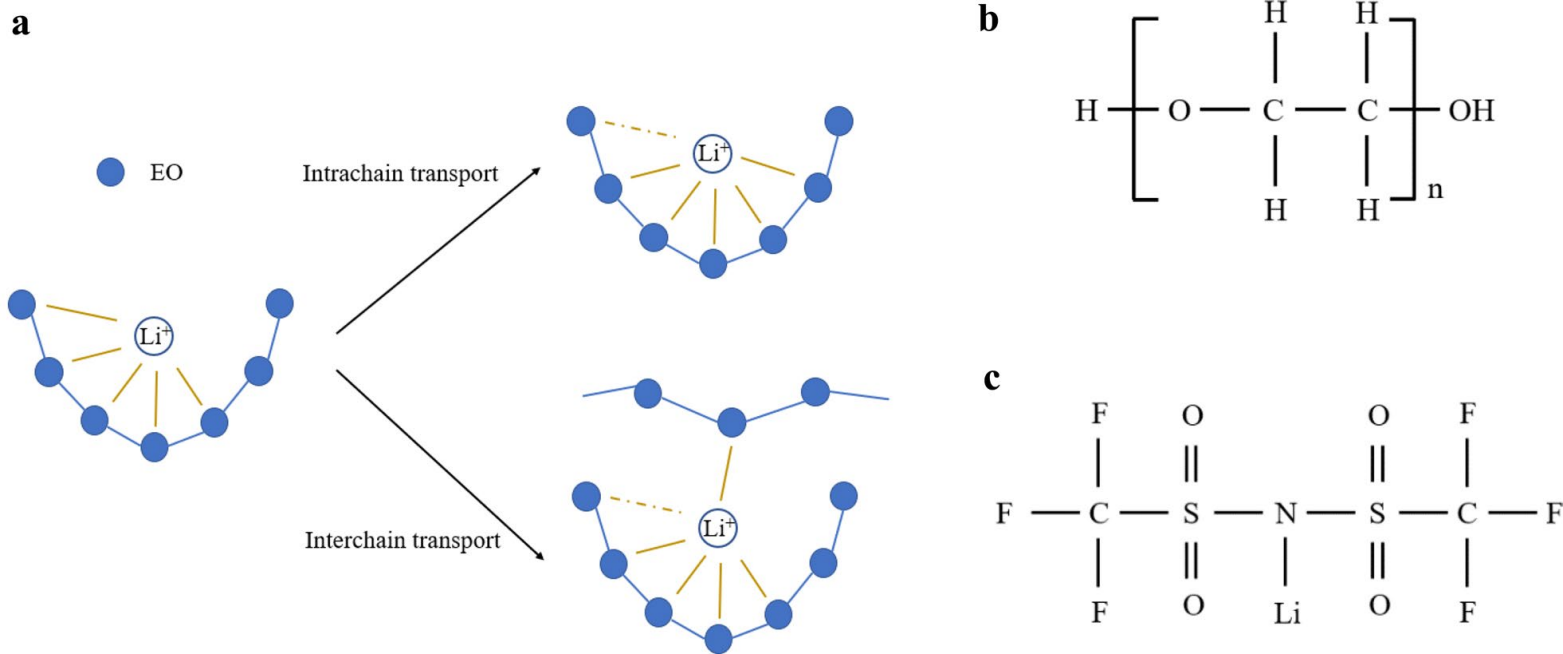
**a** Intrachain and interchain transport of Li^+^ in PEO. **b** Structural formula of PEO. **c** Structural formula of LiTFSI

Polymers used in SPEs include polyacrylonitrile (PAN) [19], PS [12, 13], polymethyl methacrylate (PMMA) [20], polyvinylidene fluoride (PVDF) [21], polyvinyl pyrrolidone (PVP) [22], etc. Compared with the other polymers, PEO has many advantages due to its special structure. PEO owns strong electron donating ether oxygen (EO) groups, soft macromolecular backbone, good thermal stability and mechanical properties. As a consequence, PEO has become the most frequently studied polymer base for SPEs. The chemical formula of PEO is H(OCH_2_CH_2_)_n_OH, as showed in Fig. 1b. It is water-soluble and in semi-crystalline state at room temperature. Usually, PEO is the name for the polymer with a molecular weight (*M*_*n*_) greater than 20,000. When the molecular weight is less than 20,000, it is called polyethylene glycol (PEG).

In SPEs, both anions and cations participate in the process of ionic conduction. However, due to the mechanism of redox reaction, only the charges carried by Li^+^ are valid. The number of evaluating the contribution of Li^+^ to the whole ionic conductivity is named transference number (*T*_*Li*+_). This number is normally below 0.5 in SPEs [10]. In the contrary, anions are fixed to the polymer skeleton through covalent bonds in polyelectrolytes. In this way, polyelectrolytes are single ion-conducting electrolytes and their *T*_*Li*+_ is close to 1. However, because of the insufficient dissociation of Li^+^, the ionic conductivity of polyelectrolytes is much lower than that of normal SPEs [23]. So, this article mainly focuses on normal SPEs instead of polyelectrolytes.

The conductive mechanism of PEO-lithium salt (LiX) matrices is shown in Fig. 1a. The whole process can be summarized by the combination and fracture of EO-Li bonds. First, the strong electron donating group EO will complex with the charge carrier Li^+^. There are about 5 EO to match with 1 Li^+^ [24, 25]. Then the conduction of Li^+^ is completed through the segmental motion of PEO molecular chains [26]. In this way, Li^+^ can be transported on a single chain or between different chains. Since the molecular chain movement is restricted to amorphous regions, the transport of Li^+^ in PEO is also limited to these regions, which means the crystallization zone won’t exhibit ionic conductivity. Therefore, the ionic conductivity of PEO largely depends on the proportion of amorphous regions. On the other hand, glass transition temperature (*T*_*g*_) is the threshold when segmental motion begins, the lower *T*_*g*_ is, the higher ionic conductivity PEO will exhibit at room temperature. As a consequence, an ideal PEO material should satisfy at least two conditions: high amorphous proportion and low *T*_*g*_. PEO with low molecular weight meets these requirements, and thus has relatively good ionic conductivity. However, the thermal stability and mechanical strength of SPEs using low molecular weight PEO as polymer backbone are terrible. With the increase of *M*_*n*_, the regularity of backbones in PEO goes higher, which leads to the reducing of the proportion of amorphous region, the increasing of *T*_*g*_ and viscosity. That means the mechanical strength of PEO is increasing while the ionic conductivity is reducing as *M*_*n*_ goes larger. In other words, PEO with low molecular weight is like liquid, while that with high molecular weight behaves like solid. The frequently used way to improve the performance of PEO include grafting to construct comb-like structure, inserting to make block structure and crosslinking [27–29]. Basically, these three methods are all trying to increase the proportion of amorphous region by destroying the regularity of PEO chains. In this way, the polymer base can not only retain the good mechanical strength of the macromolecule backbones, but also obtain relatively good ionic conductivity and low *T*_*g*_ from the small molecules [30].

Lithium salts also have a great impact on the whole ionic conductivity of PEO-LiX matrices. Generally used lithium salts include LiBF_4_, LiAsF_6_ and LiClO_4_ [24, 31]. Lithium bis(trifluoromethanesulfonyl)imide (LiTFSI), as a new generation of LiX, has particularly excellent performance in SPEs [32]. This is mainly due to its outstanding solubility. The chemical formula of LiTFSI is Li(CF_3_SO_2_)_2_N, and its structure is shown in Fig. 1c. The large TFSI^−^ anion is delocalized charge and has weak bond to Li^+^. Consequently, the dissociation of LiTFSI in SPEs is sufficient and thus more Li^+^ become free form the bondage of anions. These free-Li^+^ will then couple with EO to realize the transport in bulk PEO [33–35].

Since Li^+^ transfers in PEO by the segmental motion of molecular chains in amorphous region, the ionic conductivity of SPEs is greatly affected by temperature. As temperature goes higher, the proportion of amorphous region increases continuously, and the total transition of PEO from crystalline to amorphous is completed at melting point (*T*_*m*_), which is directly reflected by the appearance of inflection point of ionic conductivity near *T*_*m*_. Besides, Li^+^ moves on the molecular chain by complexing with EO groups. So, the ratio of EO/Li directly affects the binding and the velocity of Li^+^ transport. If EO/Li is too low, the Li^+^ carriers will be not enough in the electrolyte. If EO/Li is too high, the concentration of Li^+^ will be limited. For both situations, the ionic conductivity of PEO-LiX matrices is restricted. Generally, the highest ionic conductivity can be obtained when EO/Li = 12–16 [36–39]. Moreover, polymers’ end groups can affect the transport of Li^+^ by their different electrochemical properties.

**Fig. 2:**
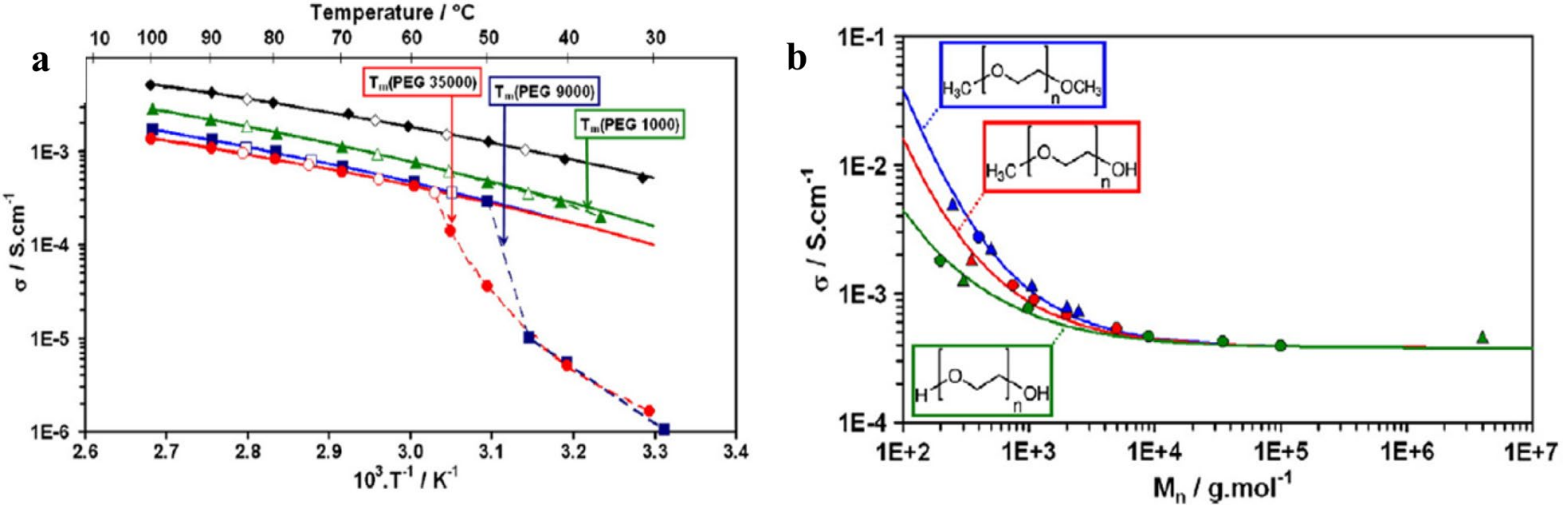
**a** Ionic conductivities as a function of temperature in different PEO-LiTFSI systems (black curve-PEG 200, green curve-PEG 1000, blue curve-PEG 9000, red curve-PEG 35,000). **b** Ionic conductivities as a function of *M*_*n*_ in different PEO-LiTFSI systems (green curve-PEG, red curve-poly(ethylene glycol)methyl ether (PEGM), blue curve-poly(ethylene glycol) dimethyl ether (PEGDM)). (Reproduction with permission from [42], Copyright © 2012 Elsevier B.V.)

These effects are indicated in Fig. 2a, b. It can be seen from Fig. 2a that as temperature increase, the ionic conductivity of all SPEs with different *M*_*n*_ does increase. The inflection point of low *M*_*n*_ systems (PEG 200, PEG 1000) doesn’t appear in the range of experimental temperature due to their low *T*_*m*_. PEG 9000 system has an inflection point at 322 K, so its *T*_*m*_ = 322 K. The inflection point of PEG 35,000 system is at 333 K, indicating that its *T*_*m*_ = 333 K. In addition, the ionic conductivity of low molecular weight PEO system is higher than that of high molecular weight PEO system at the same temperature. It can be found from Fig. 2b that the ionic conductivity of systems with –CH_3_ end group is higher than that of systems with –OH end group. This phenomenon is due to the formation of transient cross-linking structure between –OH group and ions, which will slow down the transport of Li^+^ [40]. In general, –OH is more reactive than –CH_3_ or –OCH_3_, which results in possible side reaction with electrolytes and poor high-voltage-resistance [41].

## Ceramic fillers

Adding inorganic fillers into SPEs can greatly enhance their performance. On the one hand, it can effectively improve the overall ionic conductivity. On the other hand, it not only maintains the excellent flexibility and ductility of PEO polymer base, but also improves the mechanical strength of the whole body and interfacial stability between electrodes and electrolytes by uniformly dispersed ceramic particles. Therefore, it is an ideal method to improve the performance of SPEs. The cycling performance of systems with typical fillers are shown in Table 1. Depending on whether the fillers contain Li^+^, they can be divided into passive fillers and active fillers.

**Table 1:** Cycling performance of systems with typical fillers

Fillers	Systems	Test condition	With fillers	References
10 wt% Al_2_O_3_	LiFePO_4_-PEO/LiCF_3_SO_3_-Li	C/5, 90 °C	125 mAh/g over 50 cycles	[43]
10 wt% LiAlO_2_	LiFePO_4_-PEO/LiTFSI/SN-Li	C/10, 60 °C	120 mAh/g over 25 cycles	[44]
20 wt% LAGP	LiFePO_4_-PEO/LiTFSI-Li	1C, 60 °C	108 mAh/g over 50 cycles	[45]
5 wt% LLTO	LiFePO_4_-PEO/LiTFSI-Li	C/2, 60 °C	123 mAh/g over 100 cycles	[46]
10 wt% LLZO	LiFePO_4_-PEO/LiTFSI-Li	C/10, 45 °C	158.7 mAh/g over 80 cycles	[47]
10 wt% LLZTO	LiFePO_4_-PEO/LiTFSI-Li	C/5, 55 °C	139.1 mAh/g over 100 cycles	[48]

### Passive fillers

Passive fillers refer to ceramic fillers without Li^+^. So, they are not capable in direct Li^+^ transport. Frequently used passive fillers include Al_2_O_3_ [49, 50], SiO_2_ [51], TiO_2_ [52], ZnO [53] and ZrO_2_ [54].

**Fig. 3:**
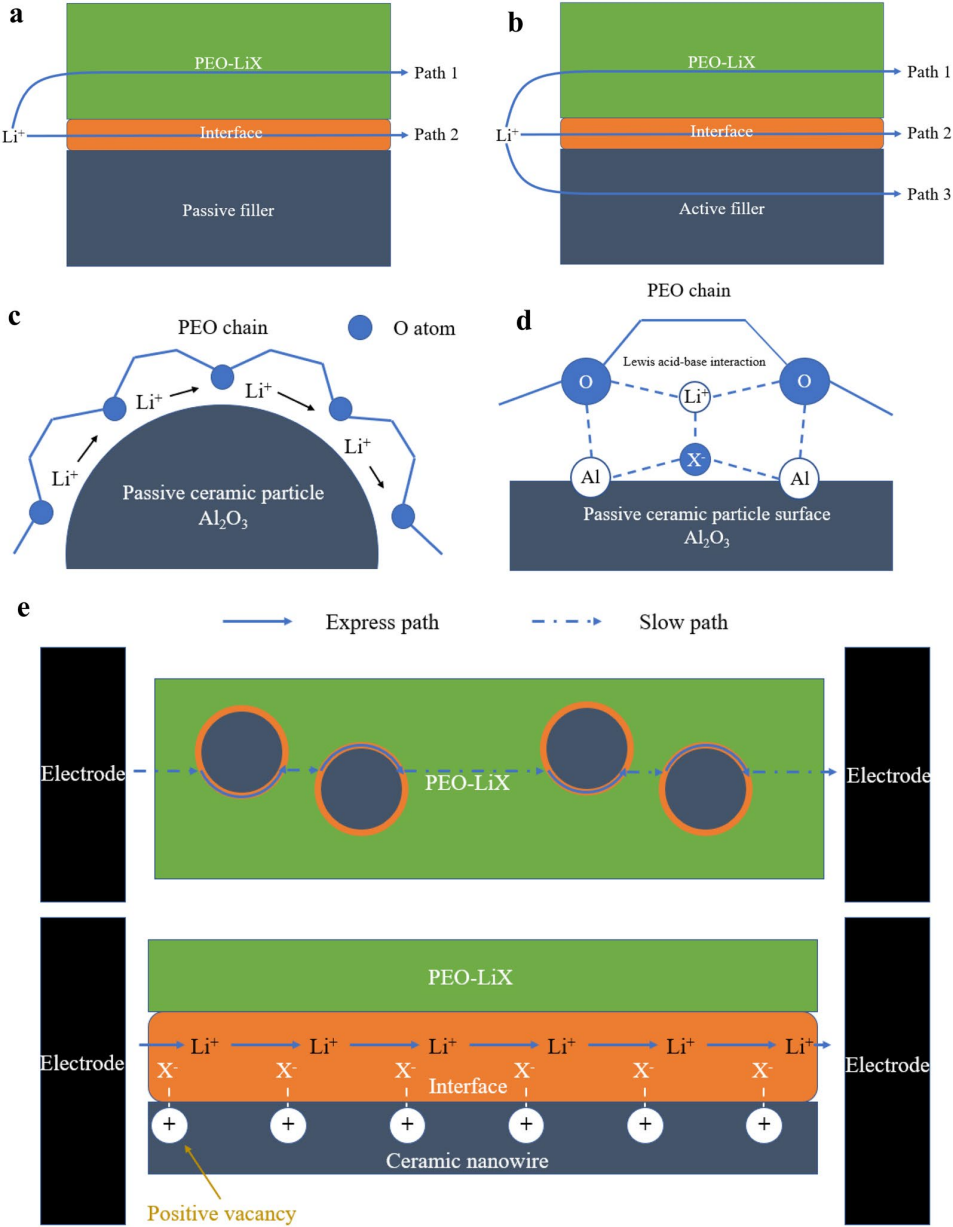
**a**Two possible paths of Li^+^ transport in PEO-passive fillers composite. **b** Three possible paths of Li^+^ transport in PEO-active fillers composite. **c** Passive fillers’ effect of distorting the regularity of PEO, which lead to the inhibition of recrystallization. **d** Lewis acid–base interaction on PEO-ceramic interfaces, including Al-X^−^, Al-O, O-Li^+^, Li^+^-X^−^. **e** Long continuous express path of Li^+^ provided by ceramic nanowires compared with discontinuous transport path of Li^+^ provided by ceramic nanoparticles

As shown in Fig. 3a, there are two possible transport modes for Li^+^ in PEO-passive filler composites. One is to transport by the segmental motion of PEO molecular chains via PEO body (path 1), and the other is via PEO/passive filler interfaces (path 2). Since fillers can greatly improve the ionic conductivity of SPEs, path 2 is obviously the key reason for such enhancement effect. However, due to the complex interfacial structure between polymer and ceramic fillers, the mechanism of such enhancement effect is still not clear.

**Fig. 4:**
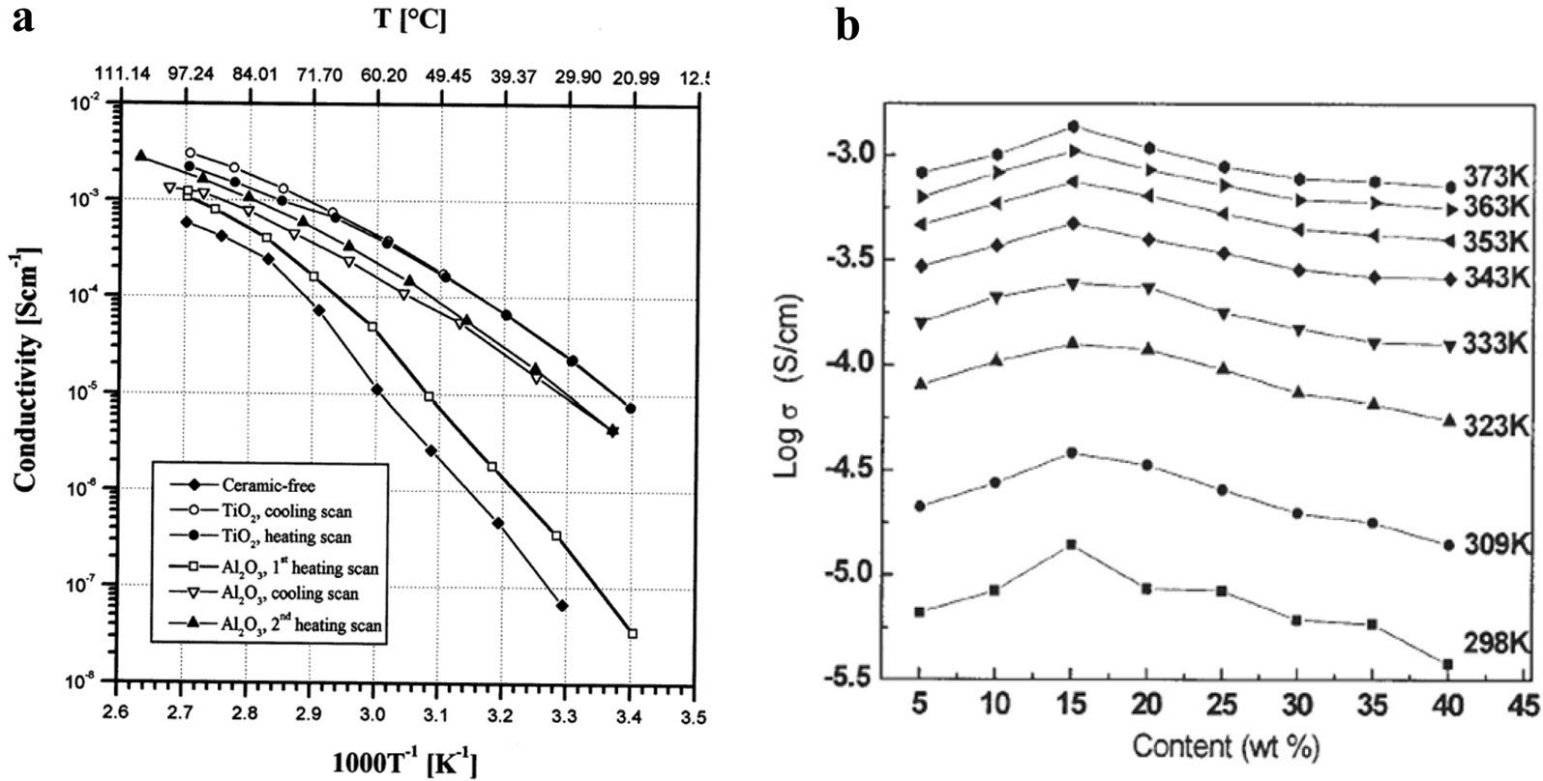
**a** Ionic conductivities as a function of temperature in PEO-LiClO_4_ systems with different fillers (no fillers, 10 wt% TiO_2_, 10 wt% Al_2_O_3_). The addition of ceramic fillers can clearly improve the ionic conductivity of SPEs. (Reproduction with permission from [60], Copyright © 2000 Elsevier Science Ltd.) **b** Ionic conductivities as a function of LATP content in PEO-LiClO_4_ systems. 15 wt% is the optimal concentration of LATP in corresponding conditions. (Reproduction with permission from [85], Copyright © 2005 Elsevier B.V.)

In 1991, Capuano et al. [55] studied the effect of γ-LiAlO_2_ on PEO-LiClO_4_ system, and identified three major functions of inorganic fillers for SPEs: improving the mechanical strength, the ionic conductivity and the stability of the phase interfaces. In 1994, Kumar et al. [56] studied the PEO-LiBF_4_-Li_3_N system. They thought that the addition of ceramics would destroy the regularity of PEO, thus increased the proportion of amorphous region. The ionic conductivity of SPEs got enhanced in this way. Besides, they proposed that the the increase of ionic conductivity might be also due to the possible formation of Li^+^ fast transport channels on the polymer/ceramic interfaces. Since the ceramic particles were dispersed uniformly in the system, such express channels would accelerate the transport of Li^+^ in the whole SPEs. In 1995, Wieczorek et al. [57] pointed out that Lewis acid center of Al atoms on the surface of Al_2_O_3_ particles would compete with Lewis acid center of alkali metal salt cations to complex with the Lewis base center of O atoms on the polymer chains. They pointed out that the addition of inorganic fillers would suppress the recrystallization process of molecular chains in PEO, which permanently increased the proportion of amorphous regions in SPEs, and thus improved the ionic conductivity of SPEs at room temperature. Croce et al. [52, 58–60] developed such theories and explained why the ionic conductivity of composite SPEs could still increase with the increase of temperature, while the whole PEO had already been amorphous when the temperature was higher than *T*_*m*_. They proposed two hypothesizes. The first hypothesize was that Lewis acid groups on the surface of ceramic fillers would compete with Li^+^ to complex with O atoms (Lewis basic group) on the polymer chains. Ceramic surface acted as the cross-linking center for PEO segments. In this way, the recrystallization of PEO was suppressed by such structure and the express channels of Li^+^ were formed. As described in detail in Fig. 4a, they found that the ionic conductivity of PEO-LiClO_4_ matrices doped with Al_2_O_3_ would not show sudden change around *T*_*m*_ and the amorphous to crystalline transition of whole PEO did not appear during the cooling process, which was hugely different from the behavior of ceramic-free SPEs and indicating the inhibition effect on recrystallization. The second hypothesize was that Lewis acid groups on the surface of ceramic particles also competed with Li^+^ to complex with X^−^, which lead to high dissociation order of LiX. These two aspects synergistically increased the concentration of free-Li^+^. Such views were further confirmed by tests. In the PEO-LiClO_4_-10 wt% TiO_2_ system [52], *T*_*Li*+_ can reached 0.6 in the 45–90 °C temperature range while in ceramic-free SPEs, such number was normally 0.2-0.3. As a consequence, when PEO has already transformed into amorphous form, these effects on helping produce more free-Li^+^ can still get stronger with the increase of temperature, which results in the slow but continuous increase of ionic conductivity. In 2016, Liu et al. [61] reported a SPE with Y_2_O_3_-doped ZrO_2_ (YSZ) nanowires on which many positive-charged oxygen vacancies were located. They found that such vacancies could help the dissociation of LiX. As a result, the ionic conductivity of the composite SPE could reached 1.07 × 10^−5^ S/cm at 30 °C while filler-free SPE could only reach 3.62 × 10^−7^ S/cm under the same condition. This experiment also verified the existence of Lewis acid–base interaction on the interfaces.

So far, the mechanism of passive fillers’ enhancement effect on SPEs’ ionic conductivity can be summarized into two types, which are showed by Fig. 3c, d, respectively.

1. Polymer-ceramic interfaces can permanently increase the proportion of amorphous region and thus increase the ionic conductivity of SPEs at room temperature by inhibiting the recrystallization of PEO.
2. Polymer-ceramic interfaces can help release more free-Li^+^ and construct Li^+^ express channels through the complicated complexation between Lewis acid center on the surface of ceramics, O atoms (Lewis base center) on the polymer chain and anions (Lewis base center) of LiX.

### Active fillers

Active fillers refer to the ceramic fillers which contain Li^+^ in their bodies, such as Li_3_N [62, 63], LiAlO_2_ [64, 65], Li_1+x_Al_x_Ge_2-x_(PO_4_)_3_ (LAGP) [66], Li_1+x_Al_x_Ti_2-x_(PO_4_)_3_ (LATP) [67, 68] , Li_3x_La_2/3−x_TiO_3_ (LLTO) [69], Li_7_La_3_Zr_2_O_12_ (LLZO) [70–72], etc. These ceramic materials own high conductivity, good chemical stability, wide electrochemical window and can directly participate into the Li^+^ transport process.

Compared with passive fillers, active fillers own stronger enhancement effect on the ionic conductivity of SPEs. This is mainly due to the intrinsic high bulk ionic conductivity of active ceramics. There are several theories to explain this phenomenon [73, 74]:

1. There are a lot of continuous defects in these structures, and the activation energy is low.
2. The ionic conductivity is achieved by concerted hopping of multiple ions instead of single ion.
3. The sublattice is highly disordered and thus the hopping between lattices won’t be disturbed.

For these reasons, these materials are also called super ionic conductors. According to the difference of structure, these materials can be roughly divided into six categories [75]: NASICON (Na super ionic conductor)-type [76], LISICON (Lithium super ionic conductor)-type [77], Perovskite-type [78], Garnet-type [79], Li_3_N-type [80] atnd BPO_4_-type [81]. Table 2 summarizes typical super ionic conductors and their ionic conductivity under room temperature.

**Table 2:** Typical super ionic conductors and their conductivity under room temperature

Categories	Typical example	Ionic conductivity under room temperature	References
NASICON (NaZr_2_(PO_4_)_3_)-type	LTP (LiTi_2_(PO_4_)_3_) LGP (LiGe_2_(PO_4_)_3_) LAGP, LATP	10^−5^–10^−3^ S/cm	[75]
LISICON (Li_2+2x_Zn_1-x_GeO_4_)-type	LZG (Li_14_Zn(GeO_4_)_4_)	10^−7^ S/cm	[77]
Perovskite-type	LLTO	2 × 10^−5^ S/cm	[78]
Garnet-type	LLZO	5.25 × 10^−5^ S/cm	[83, 84]
Li_3_N-type	Li_3_N	10^−3^ S/cm (perpendicular to c axis)	[80]
BPO_4_-type	Li_x_B_1-x/3_PO_4_	10^−7^–10^−6^ S/cm	[75]

In general, the biggest difference is that there is one more fast Li^+^ transport path in PEO-active filler composites, compared with the conducting mechanism in PEO-passive filler composites. As showed in Fig. 3b, Li^+^ can transport through PEO body (path 1), polymer-ceramic interfaces (path 2) and ceramic fillers’ bodies (path 3). In 2016, Zheng et al. [82] confirmed that Li ions were more likely to transport on path 3 by tracking the moving trail of ^6^Li^+^ in PEO-LiClO_4_-LLZO system, which confirmed that path 3 is exact the key reason to active fillers’ better enhancement effect.

### The influence of fillers’ concentration, size and shape

Capuano et al. [55] found that the ionic conductivity of SPEs can be improved by small inorganic particles, which can suppress the recrystallization of PEO molecular chains. Furthermore, they pointed out that the concentration of fillers should not be excessive, otherwise it would cause phase discontinuity problem, which led to the negative effect on ionic conductivity. Wang et al. [85–87] studied the PEO-LiClO_4_-LATP system and found that LATP fillers had a maximum concentration of 15 wt%. At this value, the ionic conductivity of the system reached the maximum value of 1.387 × 10^−5^ S/cm at 25 °C. As shown in Fig. 4b, when the concentration of inorganic fillers in the system was below this value, the crystallinity of PEO decreased with the increase of filler concentration, and the ionic conductivity increased. Such phenomena can be attributed to the enhancement effect which has been clearly described above. When the concentration was above this value, the crystallinity of PEO increased with the increase of filler concentration, which resulted in the decrease of the ionic conductivity. This may due to the crystallization site effect and molecular interaction between polymer chains, which may lead to lower mobility of Li^+^ [88]. As a consequence, the concentration of inorganic fillers is usually restricted to 10-20 wt% of the SPEs in order to get the highest ionic conductivity [89]. Reference concentrations of common inorganic fillers are listed in Table 3.

**Table 3:** Reference concentration of common inorganic fillers

Fillers	Systems	Concentration of fillers	Maximum ionic conductivity	References
LiAlO_2_	PEO-LiTFSI-15 wt% SN	10 wt%	1.36 × 10^−5^ S/cm at 30 °C	[64]
LATP	PEO-LiClO_4_	15 wt%	1.378 × 10^−3^ S/cm at 100 °C and 1.387 × 10^−5^ S/cm at 25 °C	[95]
LLTO	PEO-LiN (SO_2_CF_2_CF_3_)_2_	20 wt%	5.0 × 10^−4^ S/cm at room temperature	[87]
LLZO	PEO-LiClO_4_	15 wt%	9.5 × 10^−6^ S/cm at 20 °C and 1.1 × 10^−4^ S/cm at 40 °C	[96]

Li et al. [90] explained the influence of inorganic particles’ size on the enhancement effect by molecular dynamics (MD) simulation: lithium salts could not fully dissociate in PEO base to produce free-Li^+^. A considerable number of Li^+^ still existed in the form of ion clusters with different sizes. Inorganic fillers could inhibit the formation of such ion cluster and promote the dissociation of LiX, and thus provided more free-Li^+^. In addition, ceramic fillers’ repulsive surface could promote the movement of molecular chains and reduce the viscosity of SPEs [91], then increased the transport velocity of Li^+^. Furthermore, they pointed out that the smaller the inorganic fillers were, the stronger enhance effect they would exhibit due to the increased specific surface area. This view was also confirmed by Zhang et al. [92]. In the experiment, the maximum ionic conductivity of PEO-Li_6.4_La_3_Zr_1.4_Ta_0.6_O_12_ (LLZTO) system could increase to 2.1 × 10^−4^ S/cm at 30 °C when the particle size of LLZTO were decreased to 40 nm. Such maximum ionic conductivity could only reach 1.3 × 10^−5^ S/cm and 3.8 × 10^−6^ S/cm when the size of LLZTO were 400 nm and 10 μm [93], respectively.

The shape of fillers also has impact on their enhancement ability. Through the comparative study of PAN-LLTO electrolytes, Liu et al. [69] found that the ionic conductivity of ceramic-free SPEs was 3.62 × 10^−7^ S/cm at 30 °C, while such number in systems with LLTO particles, systems with disorderly LLTO nanowires and systems with well-aligned LLTO nanowires were 1.02 × 10^−6^ S/cm, 5.40 × 10^−6^ S/cm and 6.05 × 10^−5^ S/cm, respectively. These results indicate the importance of a continuous Li^+^ transport pathway [94], which is shown in Fig. 3e. Specifically, if the fillers’ surface orientation is close to the ideal transport direction of Li^+^, Li^+^ is likely to move quickly without disturbance.

### Preparation methods

Widely used preparation methods for composite SPEs include solvent casting [97, 98] and thermocompression [99–101]. Each method has its own advantages. For example, the membrane produced by solvent casting own good flexibility and ductility due to the residual liquid components in the body. Ceramic particles can be dispersed uniformly via the stirring process. For thermocompression, there is no organic solvent involved in the whole process, and the contact between ingredients and the air can be largely avoided. Therefore, the performance of membrane obtained by this method is more stable. Besides, this method is more convenient and time-saving. So, it is important to adopt appropriate preparation method according to different needs.

**Fig. 5:**
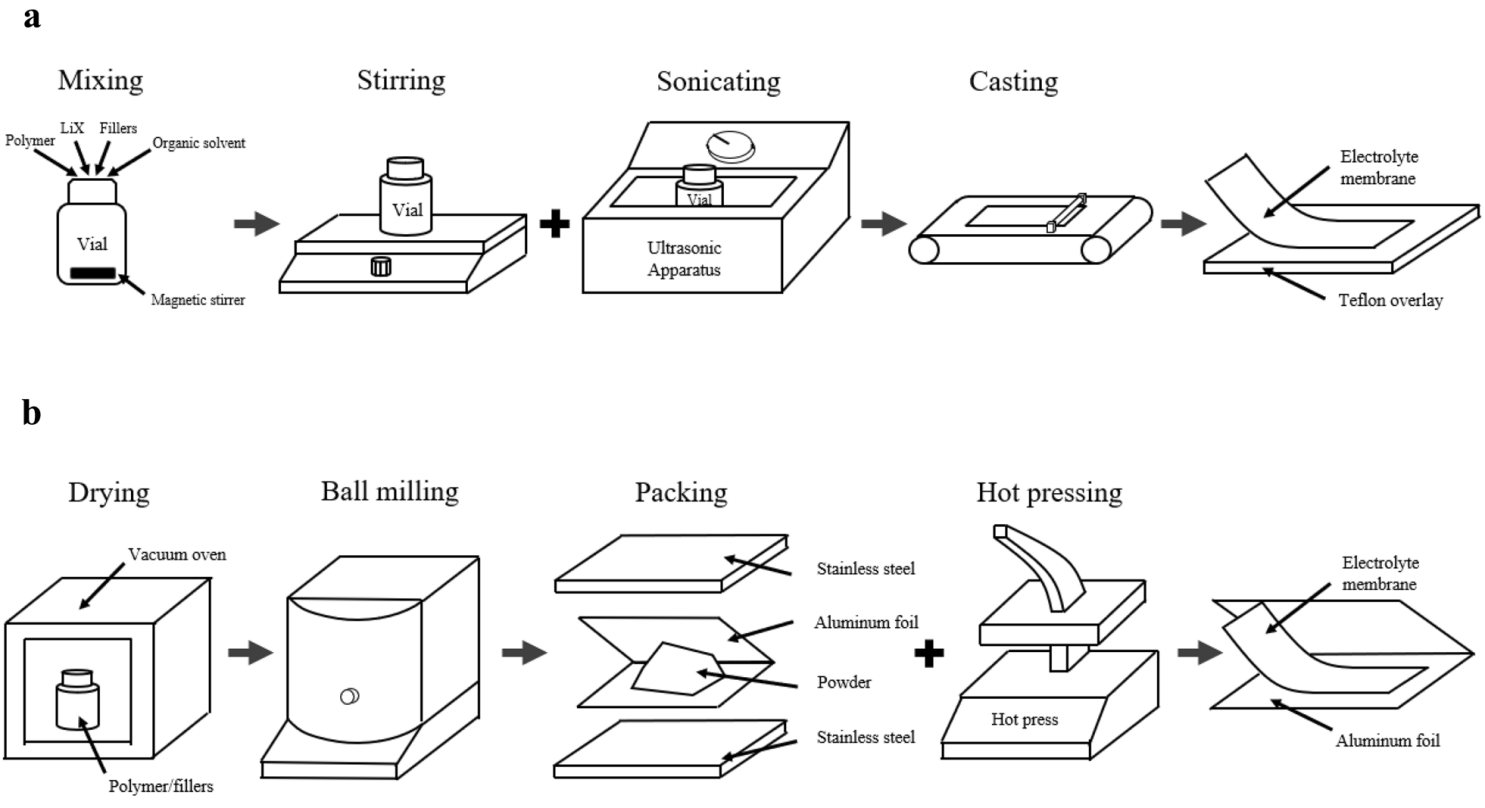
**a** Solvent casting. **b** Thermocompression

Since polymer base and lithium salts normally have good solubility in organic solvent, it is convenient to mix the components of SPEs and then cast with the help of organic solvent. This method is so called solvent casting. The basic procedure of solvent casting is shown in Fig. 5a. First, polymer, lithium salts and fillers are added into organic solvent, which is usually acetonitrile, in a certain proportion. Then, the mixture will be stirred for a long time to make sure that polymer and lithium salts are fully dissolved and inorganic fillers are dispersed uniformly. After that, the ultrasound is used to clear all the bubbles in the vial and make the composition of the mixture more uniform. Later, the colloidal solution will be casted on Teflon overlay and dried in fume hood. Until the organic solvent is totally volatilized, the membrane can be peeled off Teflon and the casting procedure is complete. Usually, the resultant film is transparent by this method.

In thermocompression, there is no organic solvent serving as medium for mixing ingredients, which is the biggest difference from solvent casting. The raw materials are mechanically mixed by ball milling, and the film is directly formed by hot pressing owing to the good flexibility and ductility of polymer. The basic steps of thermocompression are shown in Fig. 5b. First, polymer and inorganic fillers are dried in vacuum at different temperatures, due to the relatively low melting temperature of polymer. Then, sieve the ingredients to get small particles. The small particles are mixed and sealed in polyethylene bottles in a certain proportion. After that, they will be processed by ball milling to get uniform composite powder. Take aluminum foil to pack the powder and place it between two stainless steel plates and use hot press to process the powder to form membrane. After cooling, the homogeneous membrane can be easily peeled off the aluminum foil. As a result, the films obtained by this method are usually semi-transparent.

In general, the inevitable residual liquid in solvent casting can serve as plasticizer to change the overall performance of SPEs. On one hand, the introduction of liquid phase improves the ionic conductivity and ductility of the electrolyte. On the other hand, it sacrifices the strength and thermal stability because the electrolyte loses the property of all-solid-state. By contrast, the mechanical strength and thermal stability of the membranes produced by thermocompression are higher because this method is solvent-free and can greatly avoid the contact between samples and the air in the process [102].

## Conclusion

In this paper, the origin and development of SPEs have been described. The influence of *M*_*n*_, LiX, temperature, EO/Li and end groups on the ionic conductivity of SPEs is also clarified by analyzing the conductive mechanism of PEO-LiX matrices. Composite SPEs, as one of the most efficient way to improve the ionic conductivity of the whole electrolyte, is also introduced. Two possible mechanisms of polymer-filler interfaces in PEO-passive filler composites for improving ionic conductivity are clarified. Different from PEO-passive filler composites, Li^+^ can be transported through the ceramic body in polymer-active filler composites. The existence of such express path endows active fillers with stronger enhancement ability. The influence of size, shape and concentration of ceramic fillers on their ability to improve ionic conductivity are also demonstrated. Moreover, two frequently used production methods of SPEs are compared. Five conclusions can provide guidance for the preparation of composite SPEs with better performance:

1. The choice of PEO substrate. Although PEO with low *M*_*n*_ can get relatively high ionic conductivity, it is more like a liquid than a solid at room temperature, which is contrary to the initial intention of using SPEs. Therefore, *M*_*n*_ should be relatively large if allowed. Typically, PEO600k is frequently used.
2. The choice of LiX. LiX should own high solubility and high degree of disassociation in PEO, which are necessary for providing enough free-Li^+^. LiTFSI, as a new generation of lithium salt material, can meet above requirements well.
3. The choice of Li^+^ concentration. Generally, when EO/Li = 12–16, the system can obtain the maximum ionic conductivity.
4. The choice of ceramic fillers. Compared with passive fillers, active fillers provide much more Li^+^ express channels in their bodies. Therefore, active fillers are supposed to be the first choice when choosing ceramic additives. Besides, the concentration of ceramic fillers is usually restricted to 10-20 wt% to obtain the highest ionic conductivity. Moreover, the orientation of ceramic fillers is supposed to be close to the ideal Li^+^ transport path between electrodes.
5. The choice of preparation method. Thermocompression method can get rid of organic solvent and avoid contact with the air during the process, which result in more stable productions. Solvent casting method can make the ceramic fillers disperse more uniformly, and result in more ductile productions. It is necessary to choose different method depending on situations.

## Data Availability

The test materials and data are available from the corresponding author on reasonable request.

## Abbreviations

DMC: Dimethyl carbonate
EC: Ethylene carbonate
EO: Ether oxygen
LIBs: Lithium-ion batteries
LISICON: Lithium super ionic conductor
LiTFSI: Lithium bis(trifluoromethanesulfonyl)imide
LiX: Lithium salt
LAGP: Li_1+x_Al_x_Ge_2-x_(PO_4_)_3_
LATP: Li_1+x_Al_x_Ti_2-x_(PO_4_)_3_
LLTO: Li_3x_La_2/3 − x_TiO_3_
LLZO: Li_7_La_3_Zr_2_O_12_
LLZTO: Li_6.4_La_3_Zr_1.4_Ta_0.6_O_12_
*M*_*n*_: Molecular weight
MD: Molecular dynamics
NASICON: Na super ionic conductor
PEG: Polyethylene glycol
PEGM: Poly(ethylene glycol)methyl ether
PEGDM: Poly(ethylene glycol) dimethyl ether
PS: Polystyrene
PAN: Polyacrylonitrile
PC: Propylene carbonate
PMMA: Polymethyl methacrylate
PVDF: Polyvinylidene fluoride
PVP: Polyvinyl pyrrolidone
PEO: Polyethylene oxide
SPEs: Solid polymer electrolytes
*T*_*Li*+_: Transference number
*T*_*g*_: Glass transition temperature
*T*_*m*_: Melting point
YSZ: Y_2_O_3_-doped ZrO_2_
